# Supplementary material for: The polymicrogyria-associated GPR56 promoter preferentially drives gene expression in developing GABAergic neurons in common marmosets
Source: Sci Rep. 2020 Dec 9;10:21516. doi: 10.1038/s41598-020-78608-4 (PMC7726139; doi:10.1038/s41598-020-78608-4)
Supplement: Supplementary file 1 — Supplementary Information. [file 41598_2020_78608_MOESM1_ESM.pdf]

Supplementary Information

The polymicrogyria-associated *GPR56* promoter preferentially drives gene expression in developing GABAergic neurons in common marmosets

Ayako Y Murayama[1, 2], Ken-ichiro Kuwako[1, 3], Junko Okahara[2, 4], Byoung-Il Bae [5, †], Misako Okuno[2], Hiromi Mashiko[6, ††], Tomomi Shimogori[6], Christopher A Walsh[5, 7, 8], Erika Sasaki[2, 4], Hideyuki Okano[1, 2]

1. Department of Physiology, Keio University School of Medicine, Tokyo, Japan
2. Laboratory for Marmoset Neural Architecture, Center for Brain Science, RIKEN, Wako, Japan
3. Department of Neural and Muscular Physiology, Shimane University School of Medicine, Izumo, Japan
4. Center Institute of Experimental Animals, Kawasaki, Japan
5. Division of Genetics and Genomics, Manton Center for Orphan Disease Research, and Howard Hughes Medical Institute, Boston Children's Hospital, Boston, MA, USA.
6. Laboratory for Molecular Mechanisms of Brain Development, Center for Brain Science, RIKEN, Wako, Japan
7. Broad Institute of MIT and Harvard, Cambridge, MA, USA.
8. Departments of Pediatrics and Neurology, Harvard Medical School, Boston, MA, USA.

Supplementary Figure S1. Alignment of 0.3 Kbp e1m sequence of human, marmoset, and mouse. Bases that differ from human sequence are shown in black. The percentages at the end of each sequence indicate the identity to the human sequence. The 15-bp elements are enclosed in orange squares.

```
human      CCCC-ATAAA---TCGCTGTCCTAACCCCTGCCCTCCCTCCTGCCAGCTCCCTGT--CTGGCCTGGGCAGCGTCTGAGTT
marmoset   CCCG-ATAAG---TCACCATCCTAAGCCCTGTCCTCCCTCCTGCCAGCTGCCTAT--CTGGCCTGGGCAGCGTCTGAGTT
mouse      CTAGGACCCGTTCTCTCGAGTGTGAACCCAGC--TTGTCCCTGCTAGTAACCTGCTTTAGCCCCAGGCAGCCTCAGGACG

human      GA-GG------ACTTGGGAACAGGACAAGTTACGGAGCCACGTTGCTTTGCTGGGTCTGAGCCGGGGTGTGACGTAGTCC
marmoset   GA-GG------TCTTGGGCACAGGACAAGTTACGGAGCCACATTGCTTTGCTGGGTCTGAGCCGAGCTGTGACGTAGTCC
mouse      AAAGCTCTCACGCTTGGGTACA-GACAAGTTGGAGAGCCC----G---CGC----TGTAAGCCAGGCAGTGACGCAGTCC

human      CTGCAGCTGCCAACGGTTGCCAGGGCAACGGTTGCCAGGGGCTGCTGTCACCTGCGCCCCTTCT------CCCGCGCTGG
marmoset   CTGCAGCTGCCAACAGTTGCCAGGGCAACGGTTGCTAGGGGCTGCTGTCACCTGCGCCCCTTCT------CTGGAGCTGG
mouse      ATGCAGCTGCCAACGGTTGCCAGGGGAACGGTTGCCAGGGGCTGCTGTCACCTGCGCCCCTTCCTCCCCCTTGGCGCTGG

human      CGGCTGGGGCTTCTCAGCCTCTATTCCCTGGCTGTCCCCTTTGTTTGAAGCTCCAGTGAGGGAGCAGTGGCTGGG-----
marmoset   CGGCTGCGGCTTCTCAGCCTC--TTCCCTGGCTGTCCCCTTTGTTTGGAGCTCCAGTGAGGGAGCAGTGGCTGGG-----
mouse      AGGCCCGGGCTTCTCAGCT-CAGCTTCCCGCCTGCCCCCTTTGTTTGAAGCCCTAGTAAGGGGATGGTGACTGGGGGGGG

human      --GTG------GCCCAGCTTCAAAGT   100.0%
marmoset   --GTA------GCCCAGCTTCAAAGT    92.4%
mouse      GGGTAAGGGGGACCCAGGCTTTCATT    62.1%
```

Supplementary Figure S2. **(A)** Bright field images of marmoset early embryos cultured in vitro for 2 **(a)**, 4 **(b)**, 6 **(c)**, or 7 **(d)** days after lentivector infection. (e) The image of green fluorescence field of (c). *hGPR56 e1m* promoter did not work 7-day cultured early embryos. Scale bar = 100 μ m. **(B)** Genome integration analysis by fluorescence in situ hybridization. The karyograms were prepared from the peripheral blood cells of each founder marmosets, I651TgF and I757TgM. Two sets of the chromosomes from each marmoset are shown as the representatives; #005 and #007 from I651TgF. #0008 and #022 from I757TgM.

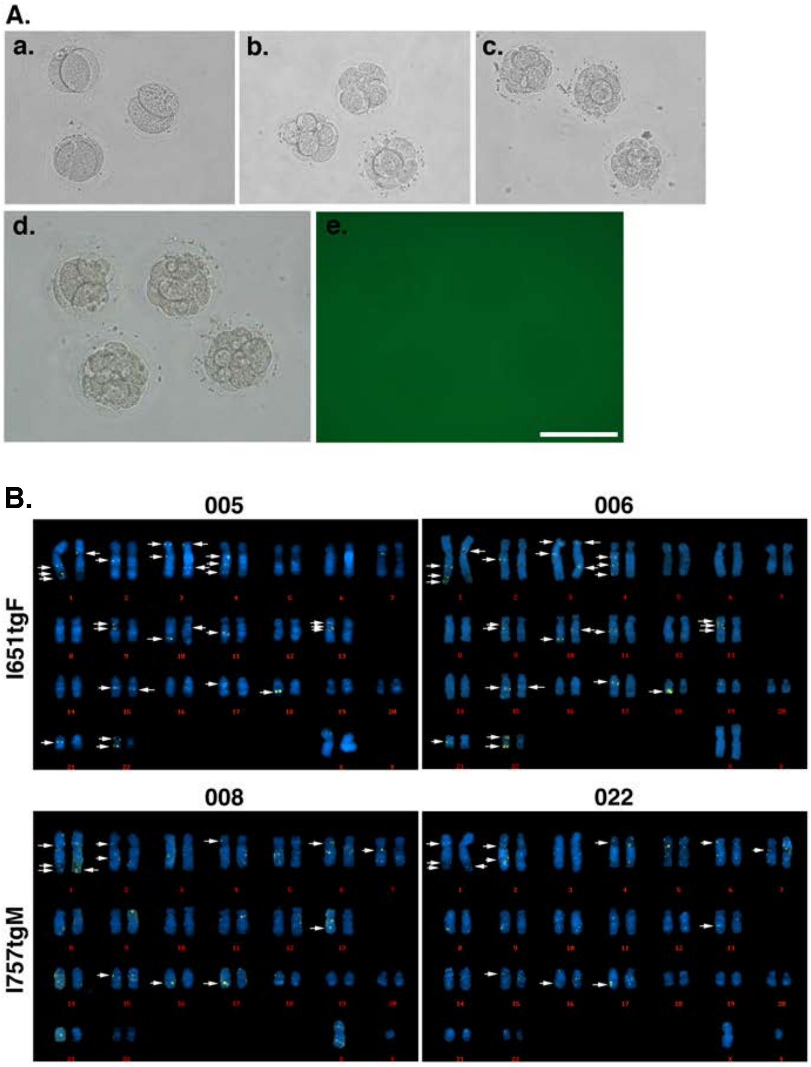

Supplementary Figure S3. (A) Brains of the E95 fetuses (no.2 and no.3) derived from I651TgF were observed for EGFP fluorescence. Lateral view of the whole brain is shown. Frontal lobe (FL), temporal lobe (TL), cerebral cortex (Cx), midbrain (MB), cerebellum (Cb) and olfactory bulb (OB) are indicated. Scale bar = 1 mm. (B) EGFP fluorescence (green) and immunohistochemistry for GFP (magenta) of the cerebral cortex at E126. DNA was counterstained with Hoechst (blue). Scale bar = 50 μm. (C) Detection of genomic integration of transgene by genomic PCR. Genomic DNA prepared from sperm of I757TgM or wild-type marmoset ES cells (negative control), and pEGFP plasmid (positive control) was used as a template.

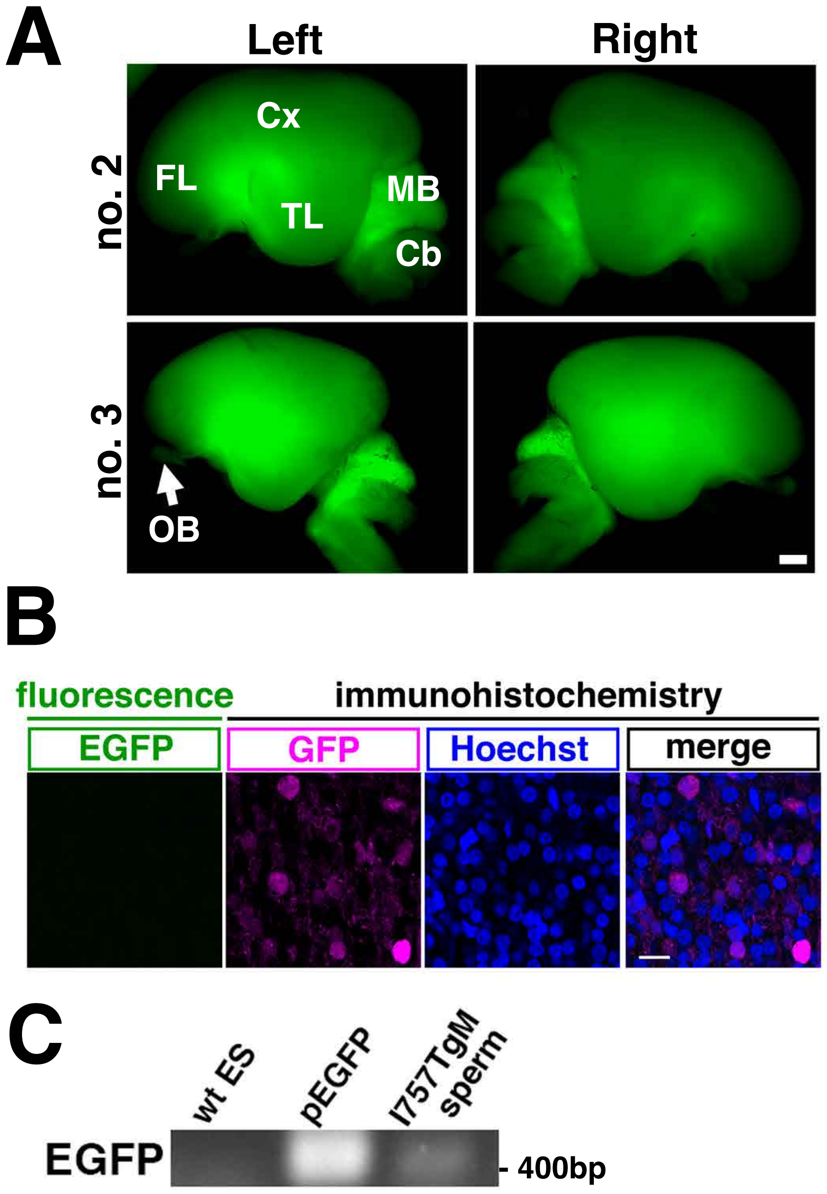

Supplementary Figure S4. Expression of *GPR56* mRNA in the brain of wild type marmoset embryo. Coronal sections of marmoset brain at the (A) 10th and (B) 12th embryonic week (EW) are hybridized with anti-sense probe for *GPR56* mRNA. Scale bar = 1 mm. Lines on the schema indicate the position of each section.

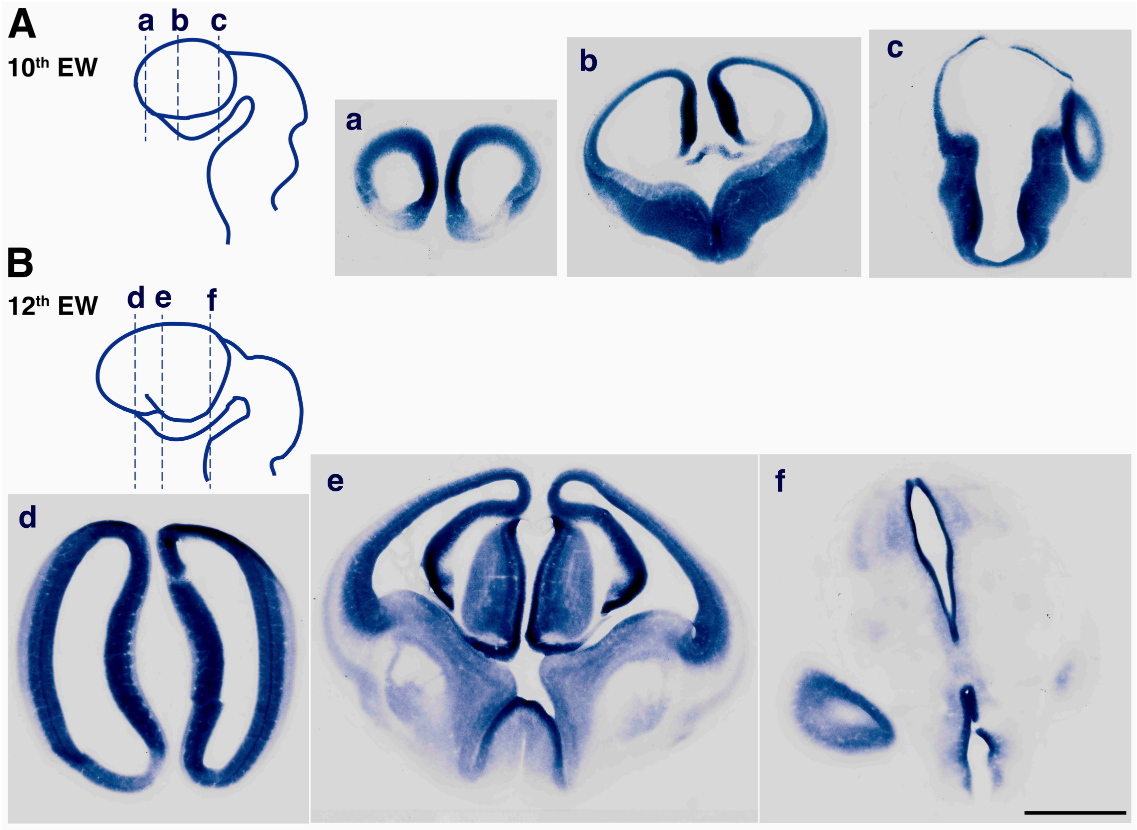

Supplementary Figure S5. COS cells transfected with HA-tagged marmoset GPR56 expression vector were fixed and stained with anti HA-Tag antibody (magenta), anti pan-GPR56 antibody (green), and Hoechst (blue). Scale bar = 50 μm.

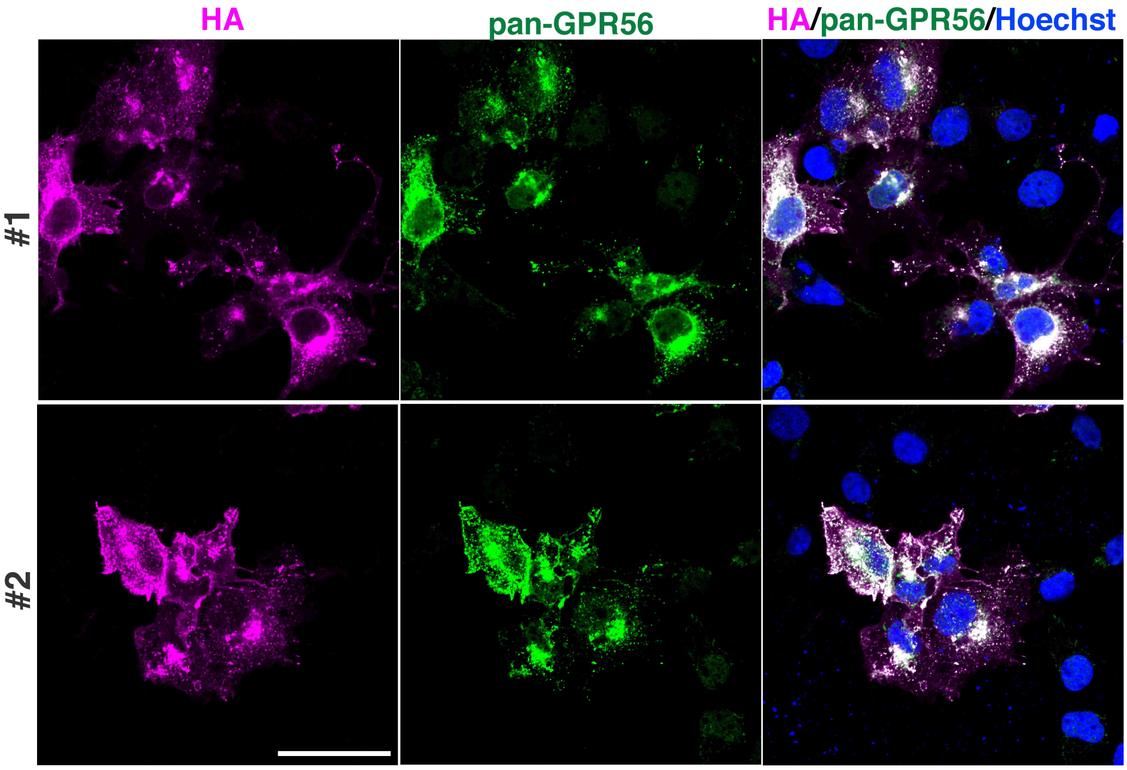

Supplementary Figure S6 (related to Figure 4C). (A) Orthogonal views of *hGPR56 e1m*-EGFP positive (magenta) and pan-GPR56 positive (green) cells in Figure 4C, excluding the one whose orthogonal view image is shown in Figure 4C. Upper (a) and Lower (b) pannels correspond to the Figure 4C a and b, respectively. The left most panels are identical to the GPP panels shown in Figure 4C, but e1m EGFP positive cells are numbered. The cell No. 1 is identical to the cell marked by the arrow in Figure 4C. Z-stack position of each orthogonal view panels is shown in parenthesis. Asterisk indicates blood vessel. (B) Serial z-sections showing the expression of each marker. Orthogonal views and serial z-sections were obtained using ZEN 2009 software (version: 6.0.0.303, Carl Zeiss, Oberkochen, Germany). Scale bars = 10μm.

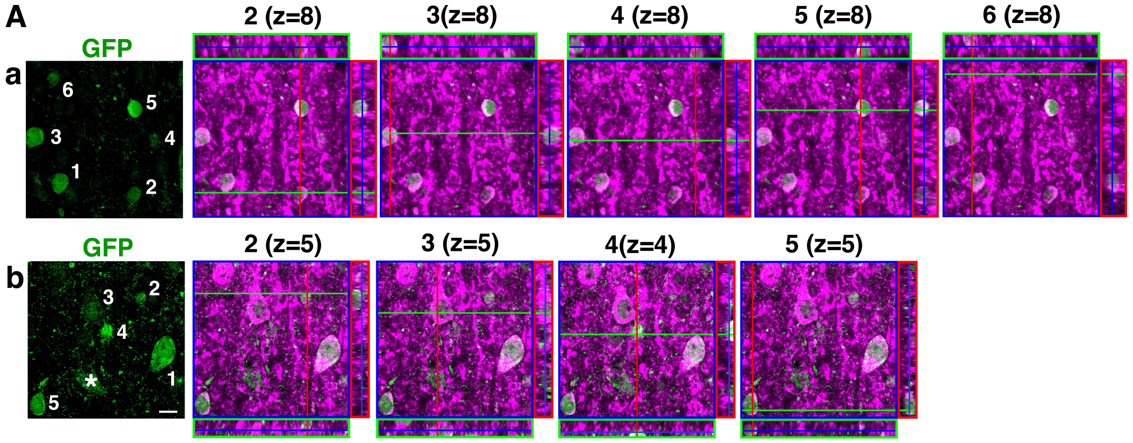

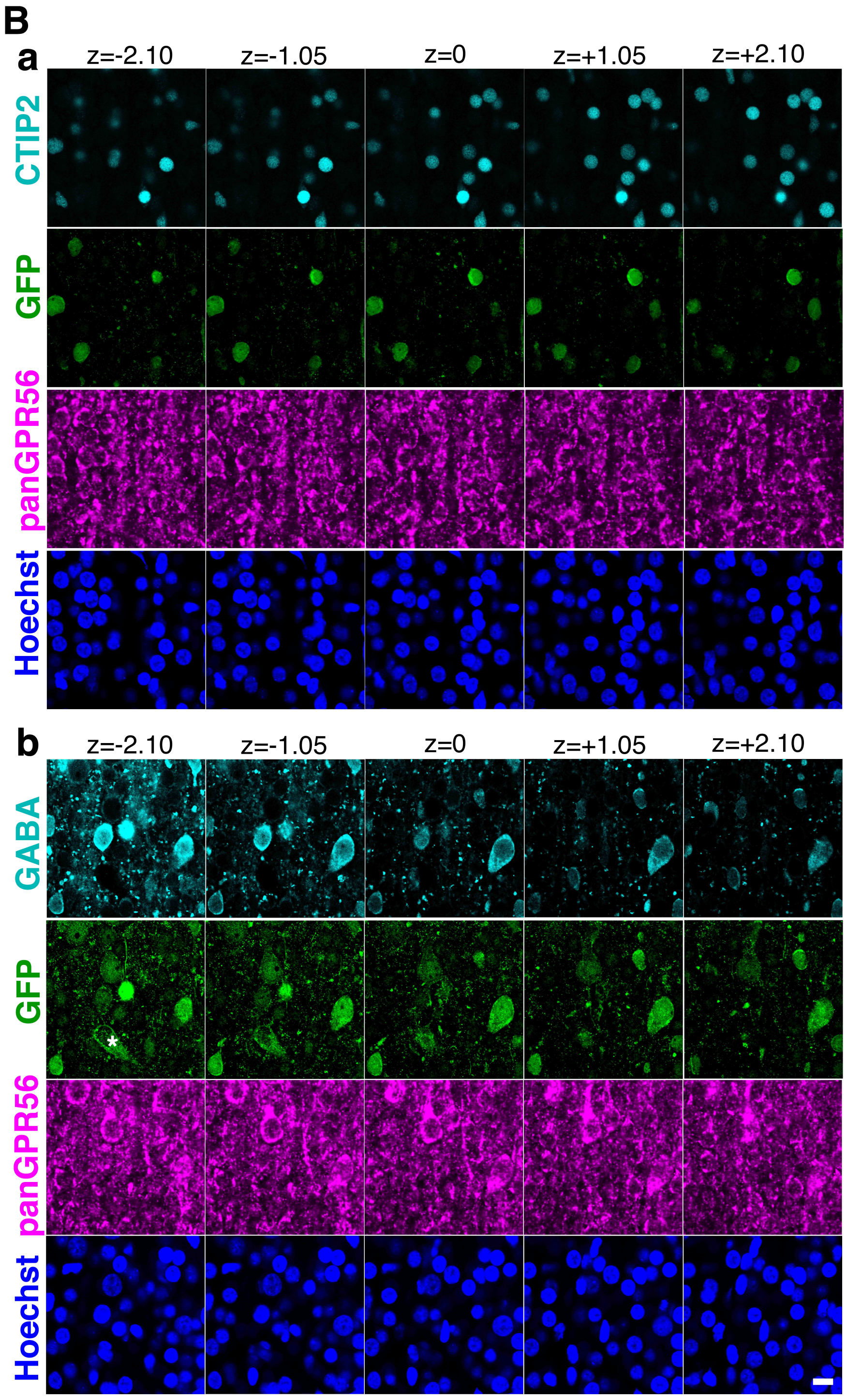

B
a
z=-2.10
z=-1.05
z=0
z=+1.05
z=+2.10
CTIP2
GFP
panGPR56
Hoechst
b
z=-2.10
z=-1.05
z=0
z=+1.05
z=+2.10
GABA
GFP
*
panGPR56
Hoechst

Supplementary Figure S7. (A) Ratio of $GABA^+$ cells among the *hGPR56 e1m*-driven $EGFP^+$ (green) or pan-$GPR56^+$ cells (magenta) in each layer of the cerebral cortex of transgenic marmoset no.1 (dark colors) and no.2 (light colors).

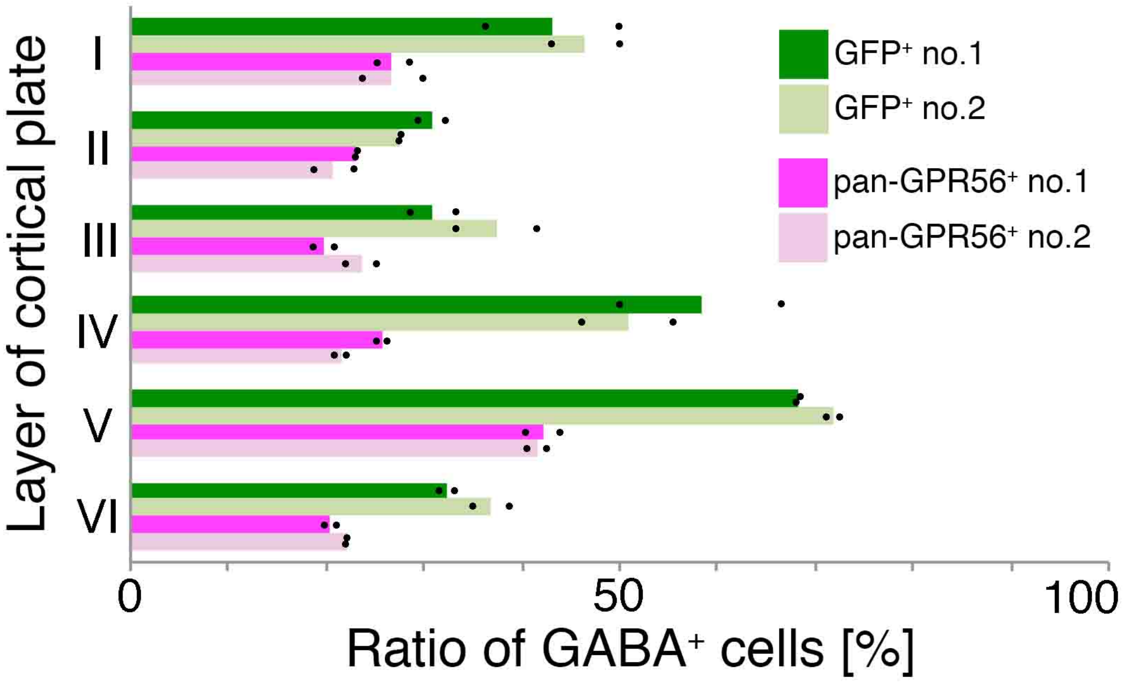

Supplementary Figure S8. (A) Immunofluorescent staining of coronal section of the marmoset brain at E89 for Nkx2.1 and GPR56. Cerebral cortex (Cx), stratum (St), presumptive lateral ganglionic eminence (pLGE) and presumptive medial ganglionic eminence (pMGE) are indicated. Scale bar = 200μm. (B) Migrating neurons expressing *hGPR56 e1m*-driven EGFP (arrows) in the cerebral cortex of the transgenic marmoset at E95. Subventicular zone (SVZ), intermediate zone (IZ) and caudate (Cd) are indicated. Enlarged image of the area marked by a square in panel a (scale bar=50µm) is shown in panel b (scale bar=100µm).

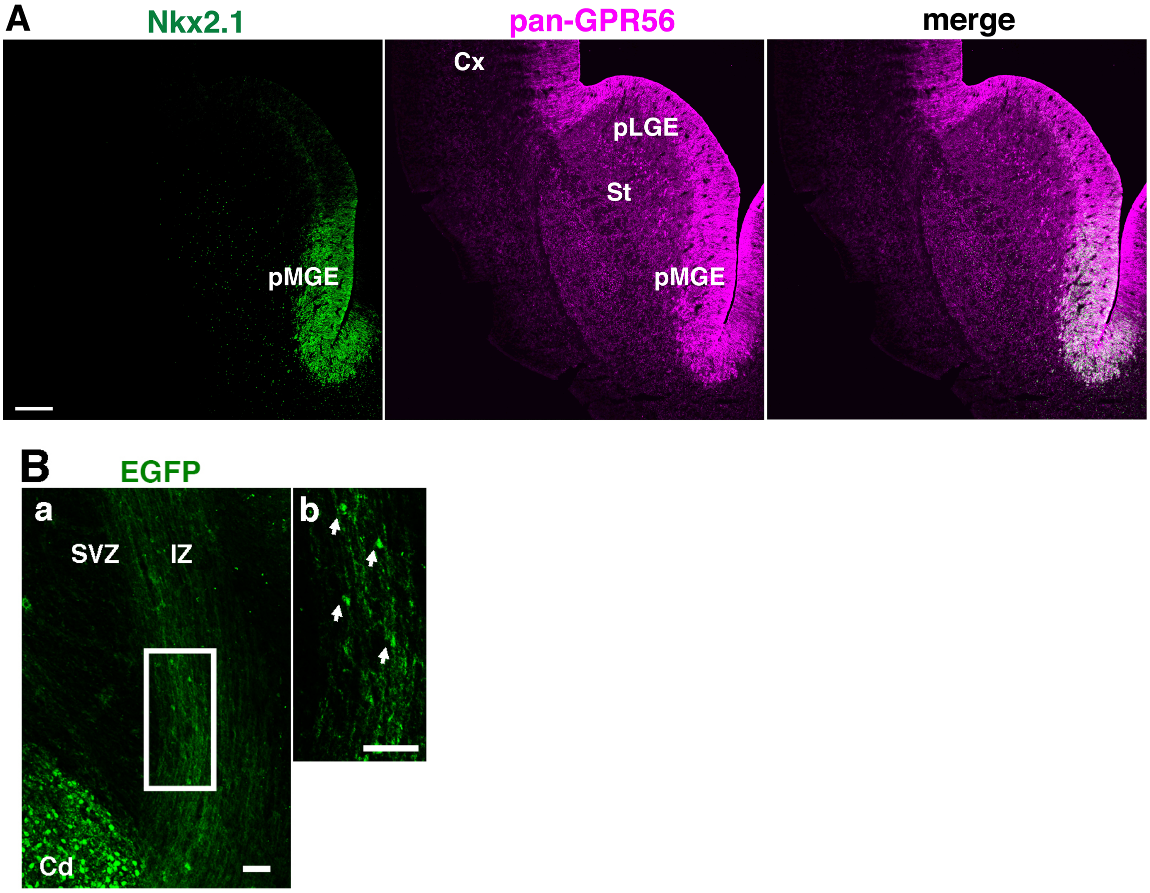

Supplementary Figure S9. **(A)** Gels scan data of Fig. 1C **(a)** and 1D **(b and c)**. **(B)** Gel scan data of Fig. S3C.

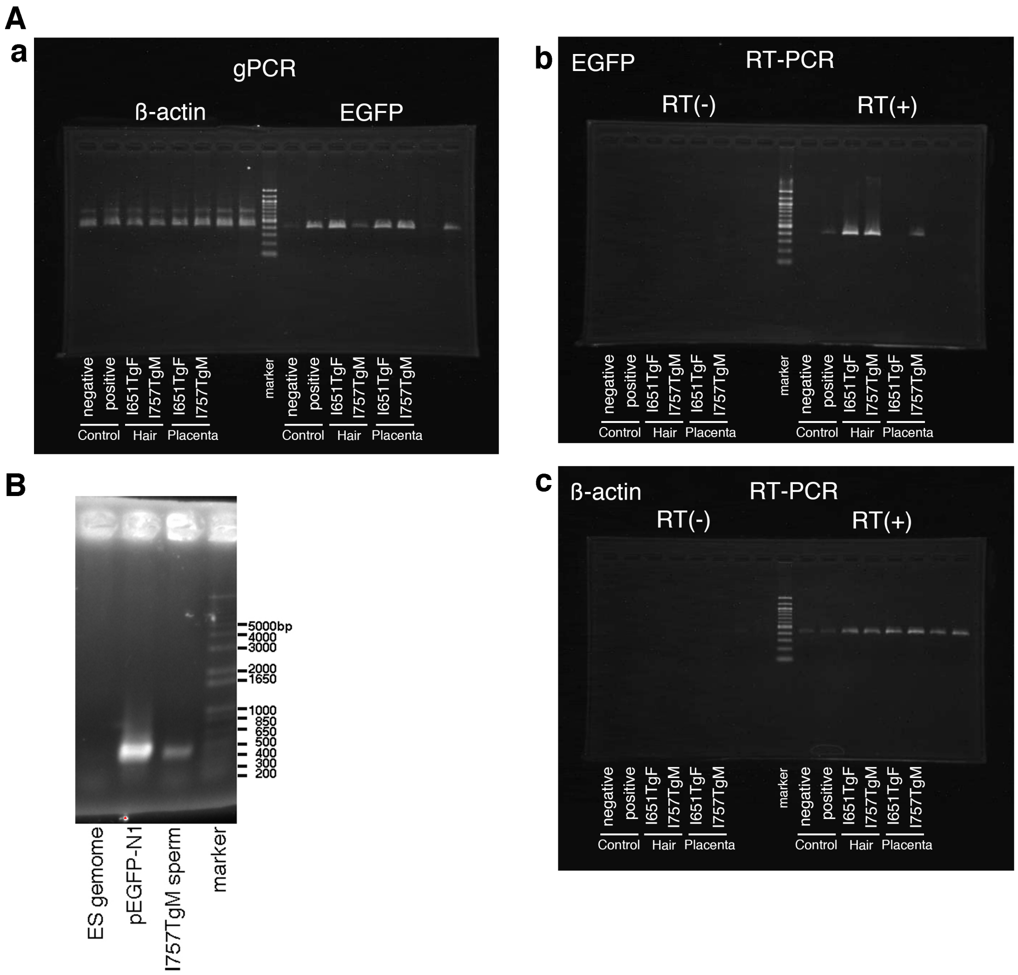

Supplementary Table S1. The percentage of *hGPR56 e1m*-EGFP+ cells in each layer.

Roman numerals are the number of each layer. The number in the parentheses is the number of sections analyzed.

individual no.		I	II	III	IV	V	VI
no.1 (12)	mean [%]	5.3	17.7	16.3	7.4	31.0	22.3
	STDEV	1.4	2.2	1.5	3.1	1.0	2.7
	SEM	0.4	0.6	0.4	0.9	0.3	0.8
no.2 (13)	mean [%]	5.7	18.5	15.7	7.0	31.5	21.5
	STDEV	2.0	2.1	2.8	2.3	1.0	2.2
	SEM	0.6	0.6	0.8	0.6	0.3	0.6

Supplementary Table S2. The percentage of CTIP2 or GABA immunolabeled neurons among the pan-GPR56 or *hGPR56 e1m*-EGFP expressing neurons in layer V. Dash means no data.

individual no.		pan-GPR56		GFP	
		Ctip2	GABA	Ctip2	GABA
no.1	% of each sections (Ctip2+ or GABA+ cells/pan=GPR56+ or GFP+ cells)	45.8 (71/155)	40.4 (59/146)	27.3 (27/99)	68.36 (67/98)
		49.7 (79/159)	37.9 (44/116)	25.3 (23/91)	68.0 (51/75)
		-		31.3 (10/32)	-
		-		30.2 (13/43)	-
	% mean	47.8	39.2	28.5	68.2
no.2	% of each sections (Ctip2+ or GABA+ cells/pan-GPR56+ or GFP+ cells)	52.6 (71/135)	40.4 (57/141)	23.5 (20/85)	71.3 (67/94)
		51.0 (50/98)	42.6 (63/148)	25.8 (16/62)	72.5 (66/91)
		51.0 (51/100)	-	28.8 (17/59)	-
		47.2 (42/89)	-	28.3 (17/60)	-
		-	-	30.2 (13/43)	-
		-	-	29.4 (10/34)	-
	% mean	50.5	41.5	27.7	71.9
total % mean		49.1	40.3	28.1	70.0

Supplementary Table S3. The percentage of GABA+ cells that coexpress *hGPR56e1m*-EGFP in each layer. Dash means no data.

layer		I	II	III	IV	V	VI
no.1	% in each sections (GABAr+ cells / EGFP+ cells)	36.4 (4/11)<br>50.0 (1/2)	32.3 (10/31)<br>29.4(5/17)	33.3 (6/18)<br>28.6(2/7)	50(5/10)<br>66.7(2/3)	68.4(67/98)<br>68.0(51/75)	33.3(10/30)<br>31.6(6/19)
	% mean	43.2	30.8	31.0	58.3	68.2	32.5
no.2	% in each sections (GABAr+ cells / EGFP+ cells)	50.0 (3/6)<br>42.8(3/7)	27.8 (5/18)<br>27.6(8/29)	41.7 (5/12)<br>33.3 (7/21)	55.6(5/9)<br>46.2(6/13)	71.3(67/94)<br>72.5(66/91)	35.0(7/20)<br>38.9(14/36)
	% mean	46.4	27.7	37.5	50.9	71.9	36.9
total % mean		44.8	29.3	34.2	54.6	70.1	34.7
total counted cell number		26	85	58	35	334	105

Supplementary Table S4. The percentage of GABA+ cells that coexpress pan-GPR56 in each layer. Dash means no data.

layer		I	II	III	IV	V	VI
no.1	% in each sections (GABAr+ cells / pan-GPR56+ cells)	28.6 (4/14)<br>25(2/8)	23.3 (7/30)<br>23.1 (6/26)	18.8(6/32)<br>20.8 (5/24)	26.3(5/19)<br>25(2/8)	40.4(59/146)<br>37.9(44/116)	19.6(11/56)<br>21.2(7/33)
	% mean	26.8	23.2	19.8	25.7	42.2	20.4
no.2	% in each sections (GABA+ cells / pan-GPR56+ cells)	30.0 (3/10)<br>23.1(3/13)	18.8 (3/16)<br>22.9(8/35)	22.2 (6/27)<br>25.0 (8/32)	22.2(6/27)<br>20.7(6/29)	40.4(57/141)<br>42.6(63/148)	22.2(8/36)<br>22.0(13/59)
	% mean	26.5	20.8	23.6	21.5	41.5	22.1
total % mean		26.7	22.0	21.7	23.6	41.9	21.3
total counted cell number		45	91	115	75	551	184

Supplementary Table S5. The percentage of PV, SST and CR immunolabeled neurons among the *hGPR56 e1m*-EGFP+ cells in layer V. Dash means no data.

individual no.		**PV+**	**SST+**	**CR+**
no.1	% in each sections (Marker+ cells/EGFP+ cells)	50.0 (16/32)	20.4 (10/49)	19.4 (7/36)
		51.2 (22/43)	21.6 (11/51)	19.6 (9/46)
		-	-	23.5 (8/34)
		-	-	22.7 (10/44)
	% mean	50.6	21.0	21.3
no.2	% in each sections (Marker+ cells/EGFP+ cells)	48.8 (21/43)	18.5 (12/65)	23.9 (11/46)
		50.0 (17/34)	19.7 (14/71)	22.1 (15/68)
		-	-	21.3 (10/47)
	% mean	49.4	19.1	22.4
total % mean		50.0	20.0	21.9
total counted cell number		152	236	321

Supplementary Table S6. The percentage of PV, SST and CR neurons in layer V. Dash means no data.

individual no.		**PV+**	**SST+**	**CR+**
no.1	% in each sections (Marker+ cells/counted cells)	32.5 (37/114)	10.0 (5/50)	11.1 (7/63)
		38.7 (55/142)	9.4 (5/53)	9.9 (8/81)
		-	-	-
		-	-	9.7 (6/62)
	% mean	35.6	9.7	10.2
no.2	% in each sections (Marker+ cells/counted cells)	30.4 (35/115)	8.8 (5/57)	12.3 (8/65)
		37.0 (37/100)	8.6 (5/58)	11.1 (8/72)
		-	-	9.7 (6/62)
	% mean	33.7	8.7	11.0
total % mean		34.7	9.2	10.6
total counted cell number		471	218	405
